# Electroacupuncture for temporomandibular disorder-related pain: clinical evidence, mechanisms, and safety—a narrative review

**DOI:** 10.3389/fpain.2026.1805774

**Published:** 2026-05-22

**Authors:** Xingzhong Zhu, Miao Huang, Ping Zhang

**Affiliations:** 1 Department of Rehabilitation Medicine, Ganzhou People's Hospital, Ganzhou, Jiangxi, China; 2 Ganzhou Key Laboratory of Neurological Rehabilitation, Ganzhou, Jiangxi, China

**Keywords:** acupuncture, electroacupuncture, literature review, pain, temporomandibular joint disorder, traditional Chinese medicine

## Abstract

Temporomandibular disorder (TMD) is a common and complex disease characterized by pain, noises in the joints, and dysfunction of the mandible, significantly impacting the patient's quality of life and day-to-day functioning. Electroacupuncture (EA), which is a combination of conventional acupuncture and electric current, has emerged as an important non-pharmacologic treatment modality for the management of TMD-associated pain. In this narrative review and comprehensive synthesis, we have discussed in detail the clinical evidence, mechanisms, and safety profile of EA for treating TMD. While evidence from RCTs and SRs suggests that EA is an effective intervention for alleviating pain, its relative superiority over manual acupuncture or sham controls should be interpreted with caution due to the observed heterogeneity in study designs. Structured analysis reveals that EA not only addresses physical pain but also potentially improves emotional distress, sleep quality, and functional outcomes, though the efficacy and effect sizes are highly contingent on the specific clinical protocols and TMD subtypes included in the trials. EA may relieve pain through various mechanisms by affecting the processing of neuropathic pain involving activation of Aβ and Aδ fibers, enhancement of the local blood flow and tissue regeneration, and immunomodulation through suppression of pro-inflammatory cytokines. EA has a good safety record, with mostly minor and short-lived side effects (pain at site or ecchymosis) and no serious complications reported to date. The clinical implementation of EA, however, requires standardization of treatment protocols, adequate practitioner training, and additional rigorous studies using large-sample, multicenter trials with prolonged follow-up in order to optimize the parameters and confirm long-term benefits. In conclusion, current evidence suggests that EA is a safe and potentially beneficial integrative modality for TMD pain management. While it appears promising as part of holistic care to reduce reliance on traditional medications, further high-quality studies are required to confirm its definitive long-term superiority and effectiveness.

## Introduction

Temporomandibular disorders (TMDs) comprise a heterogeneous group of conditions affecting the temporomandibular joint (TMJ), the masticatory musculature, and related structures, resulting in pain, limited mandibular mobility, and dysfunction. TMDs are highly prevalent, affecting approximately 10%–15% of the adult population worldwide (1). The symptoms of TMDs range from myofascial pain to internal derangement of the joints, and they frequently coexist with psychological problems that greatly affect quality of life (2).

Pharmacologic treatments, physiotherapy, and the use of occlusal splints are traditional methods used in treating patients with TMD. However, these approaches are generally not very effective and can cause adverse effects (3). While conventional therapies (e.g., NSAIDs, occlusal splints) are often first-line treatments, recent clinical guidelines indicate that their long-term (operationally defined as the maintenance of therapeutic benefits at follow-up intervals of 6–12 months post-intervention) efficacy is limited by adverse gastrointestinal effects and variable patient compliance, highlighting the critical need for effective non-pharmacological alternatives such as electroacupuncture (EA) (4).

Recently, some studies have shown that EA could alleviate the pain related to TMD by modulating pain pathways or releasing endogenous analgesic substances (5). The introduction of EA into TMD management represents an attempt to provide more holistic and individualized care, emphasizing the importance of maintaining good physical and mental health in managing chronic pain syndromes. In addition, systematic reviews and meta-analyses have begun to collect evidence about the effectiveness and safety of EA for TMD, highlighting the necessity of high-quality randomized controlled studies to establish conclusive evidence (6).

The aim of this review was to provide a systematic evaluation of the existing literature regarding the use of EA for the treatment of TMD, focusing on the effectiveness, safety, and treatment modality. By compiling evidence based on recent randomized controlled trials and meta-analyses, this article seeks to provide an overview about the developments of EA in TMD pain management. We also provide a comprehensive synthesis of future research directions and clinical applications of TMDs.

### Literature search strategy

To ensure methodological transparency, a comprehensive literature search was conducted across PubMed, Web of Science, and Scopus databases from inception to January 2026. The search strategy utilized combinations of keywords, including “electroacupuncture,” “temporomandibular disorders,” “orofacial pain,” and “mechanisms.”

Inclusion criteria focused on peer-reviewed randomized controlled trials (RCTs), systematic reviews, and foundational preclinical studies published in English. Articles were prioritized for discussion if they provided high-quality evidence (e.g., well-powered RCTs) directly addressing the clinical efficacy, neurobiological pathways, or safety profiles of EA in TMD. Exclusion criteria comprised non-peer-reviewed literature, isolated case reports, and studies lacking clear diagnostic criteria for TMD.

To address conflicting evidence—particularly regarding the absolute magnitude of pain reduction—we critically appraised the methodological rigor of divergent studies. Greater analytical weight was assigned to findings from meta-analyses and trials utilizing rigorous sham controls, while areas of uncertainty arising from study heterogeneity were explicitly acknowledged in the synthesis.

### Pathological mechanisms and clinical features of temporomandibular joint disorder pain

#### Pathophysiological basis of TMD pain

TMD pain is a complex problem caused primarily by damage to the joints and their articular cartilage, disc displacement, and muscular spasms. The pathophysiology of TMD pain is multifactorial, commonly involving a combination of mechanical, inflammatory, and neurophysiological factors. Articular cartilage injury may disrupt TM biomechanics, leading to pain and dysfunction. For instance, cartilage degradation may induce an inflammatory process that exacerbates pain through release of pro-inflammatory cytokines and other mediators. Similarly, disc displacement, which is very prevalent with TMD, may cause mechanical interference during the movement of the mandible, leading to pain and limited function. Myofascial pain due to masticatory muscle spasms—often resulting from overuse, stress, or bruxism,—creates a vicious circle of pain and dysfunction that is hard to overcome (7).

It is important to understand the relationship between neuroinflammation and central sensitization, as they play critical roles in persistent TMD pain. Neuroinflammation describes inflammation within the nervous system, which is provoked by peripheral injury and/or inflammation; it may cause a state of central sensitization characterized by hypersensitivity of the CNS to sensory input, leading to hyperalgesia. It has been demonstrated that people suffering from TMD tend to show signs of central sensitization (e.g., reduced pressure pain threshold, hypersensitivity toward non-painful stimulation) (17). Central sensitization is particularly seen among patients with chronic pain (18), suggesting that chronic TMD pain may be sustained through such neurophysiological mechanisms (8).

Neuropeptides and inflammatory mediators further contribute to TMD pain. Neuropeptides, including substance P and calcitonin gene-related peptide, play a central role in the pain pathways of TMD. Neuropeptides are released during inflammation and may cause pain by increasing the excitability of nociceptors. Current understanding of central sensitization and the involvement of inflammatory cytokines such as interleukin-1β (IL-1β) and tumor necrosis factor-alpha (TNF-α) in TMD is largely derived from preclinical animal models. However, clinical studies analyzing human synovial fluid have confirmed a direct correlation between these elevated pro-inflammatory mediators and TMD symptom severity, further emphasizing the importance of inflammatory reactions in this disease. The occurrence of neuropeptides and cytokines not only perpetuates pain but also contributes to the establishment of concomitant diseases commonly observed in TMD patients, such as anxiety and depression, adding complexity to the clinical picture (9).

In conclusion, there are a variety of factors involved in the pathophysiology of TMD pain, including mechanical, inflammatory, and neurophysiological mechanisms. A better understanding of the etiology may lead to improved treatments that not only alleviate TMD pain but also address its exacerbating factors. Further work is necessary to elucidate the complex relationships among these pathways, in order to better treat TMD pain (7).

#### Clinical manifestations and diagnostic criteria of TMD

TMDs include several disorders involving pain and dysfunction of the TMJ and related masticatory muscles. Clinical presentations of TMD vary, with the most frequent signs being TMJ pain, restricted mouth opening, joint sounds (e.g., click/popping), and mastication problems. The pain is localized at the TMJ level in patients; however, sometimes, the pain spreads toward other parts of the face, such as the head and neck, leading to major impairments of daily life and psychological distress (10). The Diagnostic Criteria for TMDs provide an overall system of diagnosis, incorporating physical examination and radiographic findings. The DC/TMD is separated into two axes, where Axis I deals with physical symptoms (e.g., pain, jaw functioning) and Axis II deals with psychosocial issues which could affect the patient's perception of his/her pain or disability (11).

The diagnosis of TMD is largely dependent on clinical assessment (history taking and physical examination) to evaluate the range of movement, tenderness, and joint sounds. Radiographic images like MRI and CBCT are commonly used as visual aids in confirming the diagnosis and evaluating the anatomy of TMJ (12). MRI can be used to visualize soft-tissue changes such as disc displacement, whereas CBCT provides detailed information on bony structures, enabling evaluation of the joint as a whole (13).

TMD pain may be either unilateral or bilateral and can involve the TMJ itself as well as other muscles around the joint, leading to the development of myofascial pain syndromes. Myofascial pain is a complex pain condition and challenging to diagnose due to its similarity in presentation with other disorders (14). Along with the physical manifestations, TMD is also associated with mental health problems such as anxiety and depression, further complicating management (15).

A thorough understanding of the multiple clinical presentations, combined with standardized diagnosis criteria, is essential for effective management of TMD. Interdisciplinary treatment approaches involving odonto-medical and psychological assessments can improve understanding of TMD and result in better and more personalized treatment planning. The combination of the clinical observations, radiological findings, psychological evaluation, and patient’s history provides an overall view of the patient's condition, thus improving treatment outcomes (16).

#### Traditional treatment methods and their limitations

Pharmacologic treatment is a common first-line approach for the treatment of TMJDs, with non-steroidal anti-inflammatory drugs (NSAIDS) and muscle relaxants frequently prescribed. Although these medicines may offer some symptomatic relief, they also have major disadvantages. NSAID treatment carries the risk of various side effects, including gastrointestinal symptoms, cardiovascular events, and kidney damage, particularly with prolonged use (17). Furthermore, muscle relaxants cause drowsiness and can be addictive, complicating drug use and patient management (18). Hence, there is a demand for safer and more effective drugs or therapies capable of alleviating pain with minimal adverse effects on the human body.

Physical therapy and orthodontic treatment are also frequently used in the management of TMDs but may have limited success. Physical therapy modalities, such as manual therapy or stabilization exercises, have demonstrated improvements in pain and function, but they are often time-consuming and may not necessarily result in long-term improvement in all patients (19). Similarly, orthodontic procedures, such as occlusal splints, aim to fix the mechanical disorder of the jaw, but their effectiveness is highly variable from person to person. For example, research shows that although splinting improves joint motion and decreases pain, it does not treat the cause of TMDs and can even cause new problems, such as bad occlusion or an overload on the joint. Therefore, despite being front-line treatments, conventional therapies usually do not meet the demands of long-term patient care.

Acupuncture is considered an alternative treatment modality for reducing the patient's pain and improving function in TMD patients. Acupuncture has been shown to decrease the severity of pain and increase the QoL (6), but durability of these results is questionable. A significant number of patients have reported that their analgesic and functional benefits fluctuate, indicating that acupuncture might help but not always in a consistent manner (6). In addition, the requirement of several treatment sessions may be an obstacle for some patients, leading to challenges in treatment adherence. Therefore, while conventional acupuncture provides an alternative drug-free approach in the management of pain, more studies are needed to improve reproducibility and develop standardized treatment protocols to ensure consistent patient responses.

Overall, treatments for TMDs—including drugs and physiotherapy, and acupuncture—all have limitations of their own that may restrict their efficacy, safety, or ability to serve as permanent solutions. Side effects of medications, variable efficacy of pharmacologic agents, and inconsistent results with acupuncture highlight the need for novel, combined treatment strategies in addressing TMD. Future research must provide integrated treatment strategies that combine existing and innovative interventions, such as EA or any other modality, to optimize patient care and improve overall treatment outcomes.

### Clinical efficacy of electroacupuncture in treating temporomandibular joint disorder pain

#### Electroacupuncture versus other types of acupuncture

Recent studies have focused on comparing the efficacy of EA with conventional acupuncture and sham treatments in TMDs. A network meta-analysis based on a systematic review of 45 trials with 2,211 subjects reported a significant reduction in pain intensity favoring EA over sham treatment (MD: −1.18; 95% CI:−2.28 to −0.09) (17). The results indicated that EA was effective in alleviating TMD pain and might be more effective than other types of acupuncture. In addition, a randomized controlled trial comparing EA, body acupuncture (BA), and classical acupuncture (CA) found that although all treatments were effective in reducing pain, EA was most noticeable in improving mood and sleep quality; these outcomes reached statistical significance (*p* < 0.05) with compared with BA and CA (18). This implies that EA not only addresses the physical symptoms of TMD but also helps improve the psychological wellbeing of patients, which is an important consideration, given the chronic nature of TMD and its associated psychological sequelae.

Beyond comparative studies of EA and other types of acupuncture, the combination of WNA and EA has been reported in some articles. The effective rate and cure rate were increased when using WNA plus EA compared with acupuncture alone or drug therapy alone (6). A meta-analysis of 10 trials involving 670 patients indicated that the RRRR was 1.20 (95%CI1.20 (95%CI: 1.02∼1.42)) for WNA and 1.82 for cure rate, indicating superior outcomes compared with conventional acupuncture and other therapies. These findings suggest that combining heat therapy with EA can improve clinical effects, likely through improved circulation and muscle relaxation.

While current systematic reviews suggest that EA is a promising therapeutic option for TMD, the overall certainty of evidence ranges from low to moderate due to methodological heterogeneity. Because high-quality, double-blinded RCTs remain scarce, preliminary claims of EA's superiority over other acupuncture modalities should be interpreted with appropriate caution. In particular, it is noteworthy that the effectiveness of EA extended beyond pain relief, with greater improvements in emotional wellbeing and sleep quality compared with conventional acupuncture. Therefore, EA may be beneficial to TMD patients due to multiple mechanisms. In addition, the combination of EA and warming techniques should be investigated through clinical trials. As the body of evidence increases, EA may emerge as an important tool in treating TMD, as an alternative to traditional treatment with improvement in patients' quality of life.

#### Analysis of dosage and treatment duration of electroacupuncture for pain management

The use of EA in pain management has received much interest in the past few years, particularly in the treatment of TMD. A critical review of the literature reveals significant variability in treatment duration, with reported protocols ranging widely from 6 to over 20 sessions, challenging the commonly cited average of 8–10 sessions, during which pain alleviation is slowly achieved and often continues beyond the end of therapy. For example, a meta-analysis concluded that after several sessions of EA, there were statistically significant reductions in pain scores for patients with chronic pain disorders, suggesting a synergistic response over multiple applications to achieve maximal efficacy (20). In addition, given the slow kinetics of pain relief observed across trials, it is possible that EA does not deliver rapid analgesia but instead produces longer-term effects that emerge slowly, thus improving patient's general wellbeing.

Individualization of stimulation parameters (frequency, intensity) is an important factor influencing the effectiveness of EA in the treatment of pain. Some studies have shown that personalization of treatment parameters could significantly improve clinical outcomes. For example, altering the strength of electrical stimulation could greatly impact the analgesic effect of EA, with the stronger agents tending to provide more effective analgesia. This variability underscores a fundamental clinical tension between the drive for standardized treatment guidelines and the necessity for patient-specific personalization. Current evidence suggests that parameter selection should be directly related to the diagnostic subtype. Specifically, predominantly myogenous TMD often responds more favorably to low-frequency, continuous stimulation (e.g., 2–10 Hz) aimed at relieving muscle spasms, whereas arthrogenous TMD with joint inflammation may require alternating dense–disperse frequencies (e.g., 2/100 Hz) to maximize the release of endogenous opioids. Without standardizing these subtype-specific guidelines, heterogeneity in intervention protocols will continue to make it challenging to compare outcomes between trials. Therefore, future work should prioritize the development of standardized protocols or treatment guidelines that improve the reproducibility and efficacy of EA as a therapy option for pain relief.

Long-term follow-up trials have consistently reported a steady analgesic effect and lower rate of pain recidivism for EA compared with conventional treatment. For example, one clinical study reported that EA-treated patients maintained reduced pain levels even after the end of therapy, with a much lower incidence of pain flares than those who did not receive EA (21). This sustained response implies that EA might be able to relieve both acute and chronic pain, making it an attractive choice in treating patients who suffer from chronic pain syndromes. Moreover, the relatively low recurrence rate suggests that this treatment may be used to prevent chronic pain states, thereby broadening and strengthening its position as a key element in managing chronic pain.

To summarize, evidence from dosing and treatment-duration studies suggests that EA is typically delivered in approximately 8–10 sessions over several weeks, with individualized stimulation parameters to maximize clinical benefit. Longer-term follow-up findings, including sustained analgesic effects and relatively low recurrence rates, further support this approach. Future work needs more standardized treatments to enhance effectiveness and generalizability of EA across populations.

#### The impact of electroacupuncture on dysfunction and quality of life

EA is becoming an attractive treatment option to relieve pain and improve function in patients with TMDs. In fact, evidence suggests that EA can provide both pain relief and significant improvement in TMJ functionality and mastication ability. For example, one RCT found that patients who received EA had significantly lower pain intensity—as measured by the VAS score for pain—and improved mandibular opening and mastication ability (18). Possible explanations for such effects involve neuromodulation of central processing of pain signals and muscle contraction, improving local blood circulation, and reducing inflammation in the tissue (22). Moreover, the positive effects of EA include not only to physical recovery but also psychological aspects. EA improves patients' general quality of life by reducing the interference of TMD symptomatology in day-to-day activities through alleviation of pain and discomfort.

Moreover, EA also provides some psychologic benefits as demonstrated through improved mental state and quality of life. After undergoing EA sessions, patients reported feeling happier and less anxious, which could be due to both analgesic effects as well as physiological effects of acupuncture (23). This is especially important in those with TMD given the high rates of co-morbid depression/anxiety resulting from their chronic pain and functional impairments (17). If pain interference can be decreased, EA allows the patient to participate in daily life tasks, providing a sense of normalcy and promoting better psychological wellbeing. The utilization of standardized assessment tools, such as the Patient Global Impression of Improvement scale, has empirically validated patient-reported improvement claims, confirming EA’s impact on QoL (6).

Furthermore, the benefits of EA extend beyond pain relief and include broader functional improvements. Studies have shown that, in addition to reporting lower pain levels, patients treated with EA perform better in mandibular-related activities (e.g., eating and speaking) (24). These functional gains are clinically important because they influence patients' perceived quality of life. The complex interplay between pain and function underlines the need for an interdisciplinary treatment strategy with regard to TMDs, where EA is only one component in an overall treatment plan.

In sum, EA demonstrates significant benefits in reducing dysfunction and improving QOL in patients with TMDs. By treating both somatic and mental symptoms, EA not only relieves pain but also improves function as well as quality of life. The incorporation of EA in standard treatment regimens for TMDs might represent an important complementary therapy to traditional approaches, eventually resulting in better patient care and satisfaction. Further studies are warranted to evaluate the longer-term impact of EA on QoL and functional improvement, as well as to clarify the mechanisms responsible for its therapeutic effects.

### Discussion on the mechanism of electroacupuncture in treating temporomandibular joint disorder pain

#### Central neuromodulation and sensitization

Current evidence, largely derived from preclinical research, suggests that EA may modulate pain through complex neuroregulatory mechanisms, including the stimulation of Aβ and Aδ fibers, which are important parts of the peripheral nervous system. When EA is used, it may stimulate these nerves, potentially causing the release of endogenous analgesic substances, including endorphins and serotonin. These neurotransmitters are hypothesized to contribute to analgesia through their ability to bind with receptors within the CNS, subsequently suppressing pain signal transmission. It has been demonstrated that stimulation of these fibers results in analgesia and an increase in global pain thresholds, a useful effect for those suffering from TMJDs with chronic pain syndromes (25).

In addition, EA was found exert strong inhibitory effects against central pain pathways as well as inflammatory responses, both of which are frequently aggravated in CP. By modulating CNS activity, EA may efficiently reverse neuroplastic changes related to pain sensitization; this would be especially pertinent in TMJD, where prolonged pain may result in increased sensitivity and altered pain perception via central sensitization. Research suggests that EA could downregulate pro-inflammatory cytokines and upregulate anti-inflammatory mediators, thus decreasing the inflammatory response that is responsible for TMJ pain and discomfort (25).

Beyond its analgesic effects, EA also contributes to nerve recovery and muscle relaxation, playing an important role on relieving myofascial pain related to TMJD. Stimulation at special acupoints could improve local blood circulation and promote nutrient supply to injured tissue, facilitating healing and recovery. Furthermore, relaxation of adjacent muscles relieves stress and minimizes TMJ load, which is often implicated in pain and dysfunction. This holistic approach not only treats the symptoms but also targets the underlying causes of myofascial pain syndrome, providing an integrated approach to treat patients with TMJD (25).

**Figure 1:**
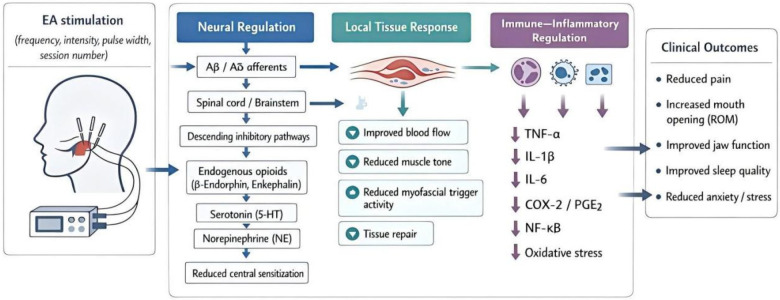
Schematic diagram of the mechanism of EA in alleviating pain related to TMDs: an integrated pathway involving neural regulation, local tissue response, and immune and inflammatory regulation.

These neuroregulatory effects of EA provide an explanation for EA’s efficacy in both alleviating pain and healing TMD. Given that EA stimulates endogenous analgesic systems while influencing both the peripheral and central processes involved in pain, it offers an attractive opportunity to improve patients' lives in this challenging musculoskeletal condition. Future studies are needed to clarify the specific mechanisms involved and to refine the treatment regimen so that it can be best utilized in clinical settings. In order to provide a comprehensive picture of EA’s analgesic effects in TMD, the major putative routes are summarized in Figure 1.

#### Peripheral anti-inflammatory mechanisms

EA has been shown to enhance local blood circulation, which plays an important role in the metabolism of inflammatory factors and the promotion of tissue repair. Improved local blood circulation can facilitate supply of nutrition and oxygen to injured tissue, thus speeding up recovery. In an experimental study about the effect of EA on chronic inflammatory pain in a mouse model, EA treatment significantly decreased pro-inflammatory cytokines such as IL-1β or TNF-α in the inflamed tissues (26). This decrease in inflammation mediators implies improvement of local tissue status, which may result in decreased pain and swelling in TMDs. In addition, EA’s modulation of local perfusion facilitates clearance of metabolic waste products and promotes the influx of immune cells essential for tissue healing and regeneration. Therefore, the local tissue response to EA represents a comprehensive strategy for pain relief and healing, indicating its therapeutic potential in TMJ disorder treatment.

Aside from enhancing blood circulation, another important function of EA is to regulate the tone of the muscles around the TMJ, which is crucial for restoring joint stability and coordinated movements. Muscle tension around the TMJ may greatly affect the joint's biomechanics, and any imbalance may result in dysfunction and pain. EA has been found to modulate muscular activity, leading to relaxation of the masticatory muscles and reduction of their hypertonicity. This modulation in muscle tone is not only effective for pain relief but also enhances the function of the joint itself by increasing its stability. By restoring a more balanced distribution of muscular forces across the TMJ, EA may improve joint position and range of motion, which are important for normal oral function. In addition, restoring muscular coordination via EA may reduce compensatory motions that commonly contribute to pain and dysfunction in TMJ disorder patients. Thus, EA’s ability to modulate muscle tone and increase joint stability is one of the main mechanisms by which it provides relief from TMJ pain.

Another important effect of EA is its impact on the metabolism of chondrocytes, which plays a critical role in slowing down the degenerative processes of the joint. Chondrocytes—the primary cells found inside the cartilage—are crucial for maintaining joint integrity and functionality. EA has been shown to stimulate chondrocyte proliferation and extracellular matrix production, which support cartilage repair (27). EA stimulation of these cells can therefore potentially reverse or mitigate the destructive pathways that lead to cartilage degradation and joint impairment seen with disorders such as TMDs. In addition, the anti-inflammatory effects of EA (reduction in inflammatory cytokines) may create an optimal microenvironment for chondrocytes. This twofold effect of enhancing chondrocyte metabolism and decreasing inflammation can reduce the progression of degenerative changes within the joint, thereby improving long-term patient outcomes with TMJ pain. In conclusion, EA's ability to support healthy chondrocytes and prevent further joint deterioration makes it an effective treatment modality for TMDs.

#### Immune and inflammatory regulation

EA has emerged as an effective treatment strategy for controlling TMDs by suppressing immune cell function and downregulating pro-inflammatory cytokines production by activating several signal transduction pathways. Research has demonstrated that EA might inhibit the secretion of pro-inflammatory cytokines such as TNF-α or interleukin-6, both of which are involved in inflammation related to TMDs (22). By targeting those inflammatory factors, EA can relieve pain and help restore immune equilibrium, thus creating a better environment for the joints. This immunomodulatory effect is critical in addressing the chronic inflammatory state that characterizes many individuals with TMD, as this chronicity may exacerbate joint destruction and associated pain.

In addition, EA has been shown to downregulate inflammatory responses occurring within the synovial membrane of the TMJ, a common suspect in TMDs. It has been reported that EA can alleviate synovitis, which involves inflammation of the synovial membrane, commonly found in patients with TMDs (22). By improving the microenvironment inside the joint, EA promotes joint function and reduces joint pain. This is especially significant since the synovium provides both synovial fluid production and nutrition to joints, and synovitis may result in additional problems, including arthralgia or decreased range of motion. That EA has been shown to ameliorate synovitis suggests that it might serve as an effective adjunct therapy to more invasive procedures used in the treatment of TMDs.

Aside from these direct effects on inflammation, EA improves general immunity as well, which is essential for the auto-healing process of our body. By enhancing immune responses, EA can potentially improve patients' ability to withstand the ongoing effects of pain and disability associated with TMDs. EA is an integrative treatment that not only treats symptoms but also promotes healing through the body itself. Taken together, these immune- and inflammation-regulatory effects suggest that EA may have therapeutic value in TMD management. By acting through multiple pathways, EA may serve as a complementary or alternative option to pharmacologic treatments, which are often associated with adverse effects (17).

In general, while preclinical data provide strong biological plausibility supporting EA's role in regulating immunity and inflammation, clinical translation remains at a preliminary stage. Current clinical evidence does not yet allow for definitive conclusions regarding its absolute immunomodulatory efficacy in human TMD patients. The capability of EA to influence immune cells, reduce inflammatory mediators, and improve joint microenvironment renders it a useful tool in the treatment of TMDs. With further research on the exact mechanism of EA, it may be incorporated into conventional treatment regimens as a promising adjunctive option. However, owing to the existing methodological heterogeneity, claims regarding EA’s absolute clinical efficacy as a definitive treatment method must remain suitably cautious pending further high-quality trials. Further research is needed to optimize parameters of treatment as well as continue exploration into how EA can improve immunity and reduce inflammation over time in patients suffering from TMDs.

### Safety and adverse reactions of electroacupuncture in the treatment of temporomandibular joint disorders

#### Reporting of adverse events in clinical studies

Safety data on the use of EA to treat TMDs have generally been positive, with most randomized controlled trials reporting good safety profiles and no significant side effects. For example, the authors of one systematic review which included 45 papers involving more than 2,200 patients noted that while adverse events were reported in 12 of these papers, none were considered serious, underscoring EA’s encouraging safety record in clinical applications (17). This is especially important given that TMD symptoms include chronic pain and functional impairments, and any good therapy must not only relieve symptoms but also ensure patient safety.

Localized side effects (pain, hematoma, and tenderness) have been reported, but these reactions are typically mild, short-lived, and self-limiting. Patients commonly experience these mild symptoms after therapy, but they are generally benign and self-resolving. For example, one trial investigating various forms of acupuncture for the treatment of TMD-associated pain concluded that there were significant improvements in both pain reduction and quality-of-life scores, with the incidence of mild adverse effects being low and tolerable (18). Overall, the benefits from EA outweigh its risks, supporting its use as an adjunctive treatment option.

Importantly, none of the included studies reported any major complications (e.g., infections and nerve injury) related to EA use. This finding is significant because many individuals avoid acupuncture treatments due to concerns about potentially life-threatening complications. As none of the included trials reported serious complications, it can be argued that EA does not pose a significant risk to the patient when applied as part of treatment regimens for TMDs. Similarly, a systematic review and meta-analysis of WNA reported no serious adverse effects, further strengthening the evidence base supporting the safety of acupoint interventions in TMD management (6).

In conclusion, existing evidence indicates that EA is a relatively safe treatment modality for TMD cases, with a low rate of AEs, which are mostly mild and temporary. This positive safety record is an important consideration for healthcare providers when choosing treatment strategies for patients with TMD, as it allows for effective symptom control while maintaining patient safety. Further studies are needed to track and report AEs, thereby solidifying the safety of EA and other acupuncture interventions for the treatment of TMD. As higher-quality data become available, adverse events should be recorded and reported systematically to better evaluate the real-world safety and effectiveness of EA and related acupuncture interventions for TMD.

#### Safety protocols and risk control

Standardized Acupuncture Treatment: Another important aspect of safety in EA relates to the use of a standardized treatment protocols along with appropriate setup of electro-stimulating parameters. One recent study established that following standard guidelines decreases the occurrence of complications associated with the procedure (17). As illustrated below, selecting appropriate needle size and length, as well as appropriate stimulation frequency, can affect both the effectiveness and safety of the therapy. It is also essential for the practitioner being trained in these techniques to avoid complications such as infections and punctures, which may arise from non-sterile environments and poor techniques. Electrostimulation parameters, such as repetition rate and energy, must be carefully adjusted in order to reduce pain/side effects without compromising on the efficiency of treatment. Several studies have indicated that improper parameter settings can lead to ineffectual therapy or aggravate painful conditions, underscoring the need for meticulous attention to detail in clinical practice (28).

Patient selection and personalized treatment design are important approaches to alleviate side effects during EA application. Patient suitability criteria for EA should include detailed patient characterization, presenting illness, and current symptomatology relevant to TMD. Treating patients on a case-by-case basis increases the likelihood of a successful outcome and decreases the potential for adverse events. For instance, patients who have certain contraindications such as bleeding disorders or infections in the area where treatment is to be performed need to be excluded from EA protocol to avoid possible injury (29). Continuing observation of patient responses during therapy enables the physician to make necessary changes during the course of therapy, thereby providing additional safety and comfort. Personalized treatment not only leads to improved therapeutic outcomes but also enhances the confidence and wellbeing of patients, both of which are key to successful complementary therapy.

It is essential for all healthcare professionals to continuously educate themselves on sterility and infection control practices, as well as proper patient monitoring during acupuncture treatment. It should also be noted that the practice of keeping everything clean while administering acupuncture treatment is paramount to good healthcare as it prevents infections and other problems. Clinicians should be familiar with established guidelines for infection prevention, including the use of gloves, syringe sterilization, and sharp waste management (30). In addition, careful pre- and post-treatment follow-up should be carried out in order to detect possible acute side effects by controlling physical parameters and evaluating discomfort and pain, which may be used to guide treatment parameter optimization. Ongoing education and adhering to proper safety guidelines will improve effectiveness of EA while promoting a culture of safety in the clinical environment, ultimately leading to better patient care (31).

Finally, EA for TMDs will remain safe if and only if proper standardized methods are followed, with personalized attention to each patient and a robust framework for continuous training and monitoring. By focusing on such aspects, clinicians will be able to minimize the risk of adverse events, while maximizing treatment benefits. As more research is published on EA, the responsibility of practitioners to ensure safety and effectiveness is high (32).

#### Patient compliance and treatment experience

Patient adherence to therapy is one of the most important factors for successful treatment and management of any disease, including TMDs. Studies have demonstrated effective pain control with EA, leading to better compliance with the therapy. EA has an analgesic effect, which has been shown to significantly reduce the patient's pain scores and improve functions compared with placebo groups (17). This significant decrease in pain motivates the patient to continue with the treatment and generates a good outlook about the therapy, resulting in improved satisfaction levels. Moreover, combining EA with other therapy methods, such as WNA or conventional acupoint acupuncture, has been associated with better results in terms of pain relief, thus enhancing patient adherence with the treatment plan (6).

Beyond physical benefits, psychological effects play an important role in shaping treatment experience and satisfaction. Psychological counseling and health education could be added into EA treatment processes to improve treatment experience. Educating the patients about the mechanisms of TMD, the possible consequences of EA, and ways of managing themselves may give them a sense of empowerment and reduce their concerns about EA. This comprehensive strategy not only improves the patient's knowledge about the treatment but also creates a sense of self-control regarding one's own health, which plays a key role in adherence (18). This can be used to address concerns/fears around the procedure itself, as well as providing motivation to the patient to complete his/her plan of care.

In the future, it is important that researchers investigate patient-reported outcomes as well as longer-term safety profiles associated with EA treatment for TMDs. Although previous trials have demonstrated the effectiveness of EA for pain relief, more research is required on the patient perspective of the care experience, quality of life, and durability or maintenance of benefit (i.e., long-term effectiveness). Such aspects should be explored to better understand how the therapy affects the life of our patients, in order to further develop individualized therapies. In addition, long-term safety assessments should be carried out, so that patients can reap the benefits of pain relief while being protected against possible harms caused by long-term EA treatment (17). By targeting these aspects, patients' engagement and satisfaction could be improved, which will eventually result in improved clinical outcomes in TMD management.

### Future research directions and clinical application prospects

Controlling the high placebo effect inherent in chronic orofacial pain therapies remains a significant methodological challenge. High-quality RCTs have addressed this by employing non-penetrating sham needles at non-acupoints. Crucially, the literature demonstrates that EA yields analgesic outcomes statistically superior to those observed in these sham groups, driven by distinct neurobiological mechanisms such as targeted endogenous opioid release.

#### The need for multicenter, large-sample randomized controlled trials

Despite encouraging clinical outcomes, the current body of literature on EA for TMD is constrained by notable methodological limitations. A significant proportion of the available trials suffer from small sample sizes, high protocol heterogeneity regarding stimulation parameters, and a lack of standardized long-term follow-up. Consequently, the absence of large-scale, high-quality, multicenter RCTs limits the ability to draw definitive, universally applicable conclusions regarding the absolute efficacy of EA, highlighting a critical gap that future research must address. For example, in one systematic review of 45 studies and 2,211 subjects, different traditional East Asian medicine treatments were rated against TMD. However, the limited sample sizes per study raise questions about the strength of these findings (17). In order to obtain stronger evidence on effectiveness, future research must prioritize larger trials, as well as diverse environments. This requirement is also supported by the fact that results from smaller trials have shown inconsistent response rates to therapy, variations in clinical practices, and lack of standardization of therapeutic regimens.

Heterogeneity among the indicators used to evaluate efficacy between and within trials hinders comparison of results. Many trials use different outcome measures, reducing the capacity to synthesize results and evaluate treatment effectiveness as a whole (6). In order to solve this problem, the development of objective criteria by which to measure effectiveness is necessary as well as the use of standardized outcome measures such as those used to measure pain, function, and quality of life. These aspects will allow easier comparison between trials and may enable more accurate meta-analyses, facilitating the creation of national treatment guidelines based on the best available research evidence.

Aside from requiring large samples and standardized evaluation metrics, we also require stratified analyses on different TMD subtypes. TMDs cover a variety of diseases with diverse etiologies and symptoms, such as myofascial pain, joint dysfunction, and arthralgia (33). It is recommended to tailor the approach to each TMD subtype for optimal individual results. For example, in one recent paper, it was suggested that individualized treatments depending on the characteristics of patient conditions are needed (34). Multicenter studies with stratified analyses should be performed by researchers to determine what treatments work best for each TMD subtype so that practitioners can tailor their approach more effectively.

In addition, multicenter studies could allow testing of different modes of therapy in a much broader scope. Several therapeutic options—such as acupuncture, physiotherapy, and pharmacologic treatments—appear to be beneficial for TMD; however, their relative efficacy has not yet been adequately compared in head-to-head studies (35). A larger, multicenter trial design will allow for comparative performance of these treatments, ultimately helping clinicians make informed decisions about treatment.

In summary, we strongly advocate for multicenter, large-sample RCTs in the field of TMD treatment. By overcoming existing shortcomings—including small sample sizes, lack of standardized assessment measures, and limited stratification by TMD subtypes—the scientific community could facilitate better and more generalizable results. This will enhance patient care by ensuring that treatments are both effective and individualized.

#### In-depth molecular biological research on the mechanism of electroacupuncture treatment

A deeper understanding of how EA regulates the neuro–immune–endocrine axis will help elucidate the mechanisms underlying its clinical efficacies in treating TMDs, as EA may exert analgesic and anti-inflammatory actions via the interplay of neurological, immune, and hormonal pathways. Recent investigations have shown that EA can modulate the secretion of different neuropeptides and cytokines, which are involved with the modulation of pain and inflammation. For example, EA can increase endorphin and enkephalin secretion, which are endogenous opioid peptides with analgesic effects (18). Furthermore, the activation of the hypothalamic–pituitary–adrenal axis has been reported, causing an increase in cortisol levels, which may decrease the stress and inflammatory response related to TMDs. This multi-targeted approach suggests that EA’s potential to relieve pain is not limited, and it can be used to restore homeostasis across the neuro–immune–endocrine system, thus contributing to the general wellness of the patient with TMDs.

While the majority of studies report positive outcomes, the overall strength of evidence is occasionally compromised by conflicting findings regarding the absolute magnitude of pain reduction. These discrepancies often stem from critical variations in control group designs—whether trials utilized waitlist controls, conventional pharmacological treatments, or non-penetrating sham needles. A critical synthesis of these trials indicates that while EA consistently outperforms waitlist controls, its comparative superiority over optimized pharmacological management requires further validation through highly standardized RCTs.

The use of contemporary imaging and biomarkers has proved indispensable for uncovering the dynamic processes underlying pain relief with EA. Contemporary imaging modalities, such as functional magnetic resonance imaging (fMRI) and positron emission tomography (PET), enable us to visualize brain activity/metabolism before, during, and after EA intervention. Such technologies have shown that EA can alter brain activity related to the experience of pain, emotions, and cognitive processes, which can give more insight into the effects of EA on CNS (17). Moreover, biomarkers for pain and inflammation, such as cytokines or neurotransmitters, can assess EA’s impact. Modern imaging techniques, such as fMRI or PET, can be used to examine brain activation patterns associated with acupuncture analgesia across individuals. These data may then be correlated with blood biomarkers measured before, during, and after acupuncture treatment sessions. The combination of modern imaging techniques and measurement of blood markers provides us with an opportunity to identify both the time course of pain relief and the underlying biological processes involved in the effects of EA. With knowledge of these parameters, it becomes possible to tailor specific treatment protocols according to the individual characteristics of each patient.

Encouraging synergy between fundamental science and clinical practice will be essential to advance precision medicine in EA therapy for TMDs. Bridging the divide between bench and bedside has potential to yield more effective therapeutic strategies and better patient care. For instance, knowledge of the molecular and cell-based mechanisms of EA can help design specific therapeutic strategies. This may include adapting EA methods to each individual patient (e.g., particular type of pain or genetic susceptibility to a particular type of inflammation) (6). Cooperation among researchers and practitioners can help ensure that findings and innovations quickly move from research to application by establishing a synergistic relationship of basic science and clinical practice so that we can move toward a more personalized and effective way of handling TMDs, ultimately leading to improved patient quality of life for those who suffer from these disabling diseases.

#### Integration of electroacupuncture with comprehensive treatment models

EA has been combined with physiotherapy, psychotherapy, and orthodontics for the treatment of TMDs. These combinations are being pursued due to the potential additive or multiplicative benefits of using two (or more) methods combined. Recent reports support the use of EA combined with physical therapy, such as manual therapy and exercise, to improve patient-reported outcomes, including pain reduction and functional restoration. EA is known for its ability to decrease pain intensity and increase jaw mobility, which may be amplified through other physical treatment modalities focusing on muscular relaxation and joint mobilization (17). Furthermore, cognitive–behavioral therapy has proven to be effective in treating psychosomatic factors related to TMDs, for example, stress and anxiety, which may contribute to pain or functional impairment. When combined with EA, psychological care may assist patients in coping with difficulties, thereby improving treatment outcomes for EA (18). Orthodontics for malocclusion correction provide structural stabilization of the TMJ, reducing loading in the joint, which is one of the factors leading to TMDs. Together, these approaches will treat not only the somatic complaints of TMD but also the psychosomatic and structural components, forming an integrated approach.

The establishment of a multidisciplinary care team (e.g., acupuncturists, physical therapists, psychologists, and dentists) can facilitate an integrated treatment plan that is adapted to each patient's specific needs. This collaborative effort provides opportunities to share knowledge and experience in order to better understand TMDs as multifactorial disorders. For instance, a patient suffering from intense pain and dysfunction can be offered an integrated approach of care with EA, physical therapy (muscle strengthening and mobility), and psychological treatment in order to manage pain as well as anxiety and stress (6). In addition, consistent cross-disciplinary meetings allow clinicians to monitor patients' progression and tailor their therapies accordingly, ensuring holistic care that addresses every aspect of a patient's illness, not just symptom relief but also improved quality of life. This approach helps patients feel more satisfied about the results of their treatment.

The future of EA is likely to involve smart devices that offer individualized and fully automated therapy by adapting electrical stimuli patterns according to the specific requirements of each patient, taking into consideration the presence of pain, prior treatment, and individual features related to TMDs. EA systems that are able to adapt the stimulation intensity and frequency based on real-time responses will allow for personalized therapy, thereby enhancing the therapeutic response while reducing the patient's pain levels (17). This is an important factor when treating TMDs, as patients' responses to treatments may be varied. Furthermore, combining telemedicine with smart EA devices may enable remote monitoring and consultation, thereby improving access to care and convenience for patients. Therefore, instead of requiring patients to attend multiple in-person visits at different locations, follow-up appointments and treatment adjustments could be provided remotely when appropriate. This will lessen the burden of the treatment process itself and give greater control to the patient. Ultimately, the integration of EA with novel comprehensive models, such as advanced cognitive–behavioral therapy or sophisticated technologies, represents a promising frontier. However, it must be explicitly noted that these combinatorial approaches remain largely experimental. Unlike standard EA protocols for pain management, which are supported by established clinical guidelines, these emerging therapies require rigorous validation in large-scale trials before they can be recommended as standard clinical practice.

#### Development and promotion of standardized treatment protocols

The development of evidence-based clinical practice parameters for EA as in TMD management is critical to ensure that all patients receive high-quality, equitable care. Clinical protocols and practice guidelines should be established to give providers clear, evidence-informed recommendation about how to use EA, including specification of treatment parameters (i.e., electrode location, frequency, time, and intensity of stimulation). These aspects have been shown to influence the outcome of treatment. Recent studies have confirmed that EA is able to decrease the pain related to TMDs, emphasizing the need for a structured method (17). Developing a proper clinical pathway would optimize therapeutic effects while minimizing side effects. In addition, integrating EA into current treatment modalities used for TMDs may aid in providing patients with a more comprehensive care plan, as it may be complemented by other modalities, including pharmacotherapy and physical therapy.

Beyond creating clinical pathways, there needs to be more emphasis placed on educating physicians and patients about EA methods. Physician training must include the theory behind acupuncture, hands-on experience in needling techniques, and nuances in using EA. The probability that a patient will be treated successfully is increased when healthcare workers are trained and knowledgeable in these skills; this will also equip clinicians to provide individualized care based on each patient's specific condition. In addition, educating the patient is also important as their compliance with therapy and their overall satisfaction depends on it. Educating the patient on the possible efficacy and side effects of EA may promote shared decision-making between the therapist and the patient, encouraging active participation in their treatment plan. It has been demonstrated that patient involvement leads to better outcomes in the treatment of other chronic pain syndromes such as TMDs (18). Therefore, a two-pronged effort involving not only providers but also patients is instrumental, as they are key stakeholders in implementing EA into clinical practice.

**Figure 2:**
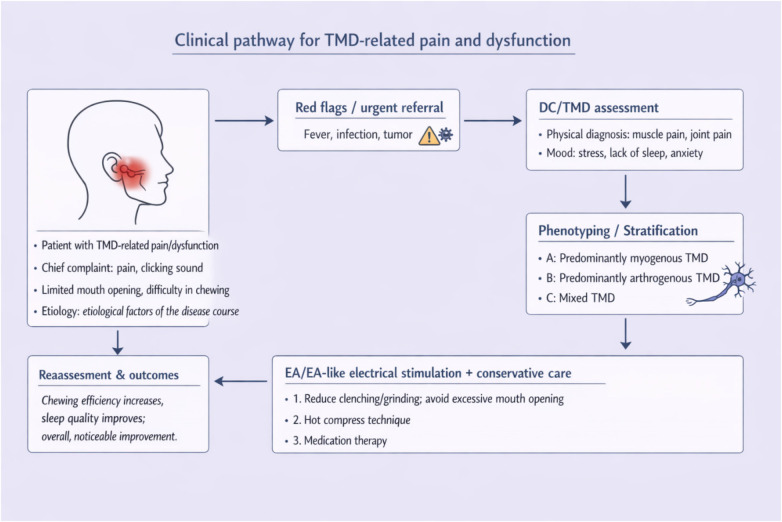
Clinical decision-making flowchart for EA intervention in patients with TMD pain.

In order to promote EA’s role in clinical practice, it is essential to include EA in clinical guidelines and treatment protocols of TMDs. If EA is incorporated into official clinical guidelines, its visibility among clinicians may increase, which could facilitate broader adoption in routine practice. This can be accomplished by publishing work showing that EA is effective in treating the pain and dysfunction associated with TMD. Systematic reviews and meta-analyses have demonstrated that different forms of acupuncture (including EA) are effective in improving symptoms for patients with TMDs (6). Presenting compelling data to relevant medical boards/guideline committees will help shape policies that encourage adoption of EA as part of routine practice. Engaging in multidisciplinary collaborations with dental, pain management, and rehabilitation experts will further strengthen the argument for EA, ensuring that it is considered an important part of the overall management plan in treating TMDs. Based on the current evidence and a phenotype-based patient assessment, we propose a pragmatic clinical workflow that includes EA as one option for managing TMD (Figure 2).

Finally, it is important to develop and promote standardized guidance for EA within TMD management, as this may improve clinical practice by supporting clinician training and facilitating the integration of EA into evidence-informed care pathways. This complex strategy is likely to improve not just outcomes but also perception and uptake of EA as an alternative therapy in the management of TMDs.

#### Economic and social benefit assessment of electroacupuncture treatment

Evaluating the economic and social benefits of EA in the treatment of TMD is essential for understanding its value compared with conventional approaches. EA has been reported to effectively relieve the pain caused by TMD, which may result in considerable gains for the patient's wellbeing and work performance. A meta-analysis found that different acupuncture modalities (including EA) had positive effects on the reduction of pain intensity and improvement of function among TMD patients, indicating the possibility of using these treatments at a lower cost than traditional options, like pharmacological treatment and/or surgery (17). The economic advantages of EA stem from reductions in both direct medical costs (ongoing treatment) and indirect costs such as lost productivity resulting from pain and disability. In treating pain and functional limitations, EA may lead to earlier resumption of employment, which will reduce healthcare and societal costs for patients.

In addition to its burden on individuals with TMD, this disorder has a significant effect on society through absenteeism from work, disability pension, and increased healthcare utilization by both employees (employers) and service providers. Studies have previously reported that workers suffering from TMD were more likely than controls to take sick leave and claim disability benefits, highlighting the need for efficient treatments such as EA (36). By mitigating the symptoms of TMD, EA can improve patients' ability to perform their daily duties, while reducing the burden on the social security system and healthcare resources. This is particularly relevant given the recent increase in the prevalence of TMD, which is becoming an increasing public health burden worldwide.

In addition to clinical efficacy, economic evaluations of EA for TMD should also consider the broader implications of improved patient outcomes on quality of life and mental wellbeing. The psychosocial impact of persistent pain may be reflected in higher cost of care due to co-morbid psychiatric conditions, such as anxiety and depression (6). By offering an effective non-invasive therapy option, EA may be able to decrease the prevalence of such comorbidities, ultimately resulting in reduced total healthcare costs and enhanced patient satisfaction. Moreover, incorporation of EA into routine clinical practice for TMD may set an example for other chronic pain syndromes, moving toward a more comprehensive, patient-based model of pain treatment and management, which is also economically efficient.

Finally, the results of economic evaluations of EA may provide healthcare policymakers with information needed to support the development of healthcare policies and decisions. For example, policymakers could use such evidence as a rationale for including EA within clinical practice guidelines for TMD, thereby ensuring patient access to efficacious, cost-effective treatment regimes. Such policy interventions may improve the sustainability of healthcare systems through reduced dependence upon costly, more invasive therapies, while also delivering better healthcare to patients. Finally, EA treatment for TMD offers multiple avenues of positive impact on both society and the economy. EA contributes directly through reduced healthcare costs, improved patient productivity, and better quality of life, thus representing an important aspect in the treatment of patients within a global approach to managing TMDs. Further research is needed to generate robust evidence, which could be used by clinicians as well as decision-makers to support healthcare policies (37).

## Conclusion

EA appears to be a promising, broadly benign non-pharmacologic adjunctive option for TMD-associated pain. Although some clinical trials suggest potential advantages over conventional acupuncture and standard treatments, the high risk of bias and heterogeneity in the existing literature preclude definitive claims of absolute superiority. Beyond analgesia, EA may improve pain-related emotional distress and sleep quality, outcomes that are closely linked to patient-reported quality of life. Mechanistic evidence suggests that the effects of EA occur via several mechanisms, such as modulating nociceptive processing, promoting local tissue recovery, and controlling immune–inflammatory signaling. These effects biologically align with the multifactorial physiopathology of TMD, which includes peripheral and central sensitization, musculoskeletal dysfunction, and inflammation. These observations allow clinicians to rationally optimize stimulation parameters and acupoint selection according to a patient’s phenotype. Despite an overall favorable safety profile, broader clinical implementation requires standardized protocols, operator training, and structured adverse-event reporting, given that technique and stimulation parameters can influence both efficacy and tolerability. Future progress will depend on well-designed, multicenter RCTs with an adequate sample size, longer follow-up, and harmonized outcomes to define effect size, durability, and relative efficacy against standard-of-care treatment. Concurrent mechanism investigations with quantifiable biological markers and electrophysiological outcomes will aid in better defining inclusion criteria and facilitate targeted, personalized EA protocols. In summary, the application of EA in a multimodal approach to treat TMD pain might lead to better results with less need for pharmaceutical treatment, provided that continued improvements in the quality of evidence and clinical standardization are achieved.
